# Renal Artery Rupture in Association With Fibromuscular Dysplasia

**DOI:** 10.1177/2324709618762585

**Published:** 2018-03-16

**Authors:** Tamer Akel, Suzanne Elsayegh

**Affiliations:** 1 Staten Island University Hospital, Staten Island, NY, USA

**Keywords:** fibromuscular dysplasia, renal artery rupture, aneurysm, dissection

## Abstract

Fibromuscular dysplasia is a noninflammatory arteriopathy of unknown etiology that affects medium-sized arteries. Although patients affected with it are often asymptomatic, some might have recurrent catastrophic events that depend mainly on the arterial bed involved. The most worrisome vascular complications of the disease are aneurysmal rupture and arterial dissection. Herein, we report a case of a 49-year-old woman who presented with sudden-onset abdominal pain without any inciting factors. She was found to have active blood extravasation from a capsular branch of the renal artery that happened spontaneously. Angiography revealed fibromuscular dysplasia in the renal arteries without any obvious aneurysms. To our knowledge, this is the first case in the literature describing such an event. In this article, we also review the possible underlying pathology behind such an event.

## Introduction

Fibromuscular dysplasia (FMD) is a noninflammatory, nonatherosclerotic arteriopathy of unknown etiology that affects medium-sized arteries. It is characterized by fibrous tissue formation within the arterial walls leading to arterial narrowing.^1,2^ Although the prevalence of FMD remains unknown, data from asymptomatic potential kidney donors revealed that 4.4% of them had FMD.^3,4^ FMD has been associated with various vascular events including aneurysms, artery dissections, and tortousities.^3^ According to the FMD Registry, the prevalence of renal artery aneurysms (RAAs) was 5.6%, while the prevalence of renal artery dissections was 4.3%.^5^ The most commonly affected areas are the renal, carotid, and vertebral arteries, but it can almost occur in any artery.^1,6^ While the underlying pathophysiology remains elusive, certain risk factors exacerbate it, mainly smoking.^1,7^ Symptoms of FMD are variable and depend primarily on the severity and location. Patients are usually asymptomatic; however, some may have recurrent catastrophic events like strokes and myocardial infarctions.^3^ The diagnosis of FMD remains challenging and is made usually by imaging studies with catheter-based angiography. Nevertheless, pathology specimens are the gold standard, but are uncommonly available in modern practice.^7^

## Case Report

A 49-year-old female without any known past medical history presented to the emergency department with 4 hours of continuous right lower quadrant abdominal pain. She has no past surgical history and has no previous medical follow-up. She described the pain as 9/10 in severity, dull, deep, and without any associated gastrointestinal or genitourinary symptoms. Aside from occasional use of nonsteroidal anti-inflammatory drugs for tension headaches, there is no other drug or herbal use. The patient’s social history was remarkable for smoking at least 10 cigarettes per day for the past 30 years. On physical examination, her blood pressure was 200/104 mm Hg, and she had severe right lower quadrant abdominal tenderness without guarding.

**Figure 1.**
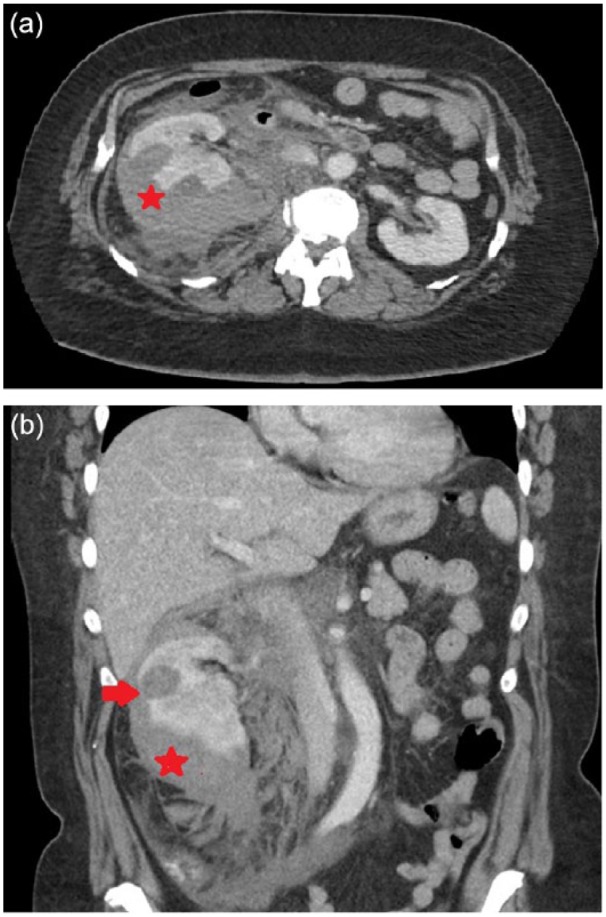
(a) Axial and (b) coronal contrast-enhanced computed tomography scan of the abdomen showing a right renal extra-capsular hematoma.

Initial complete blood count, liver function tests, coagulation studies, and lipase level were all unremarkable except for a white blood cell count of 16 × 10^9^/L, predominantly granulocytic. Blood chemistries were unremarkable aside from a creatinine of 1.23 mg/dL with an estimated glomerular filtration rate of 48 mL/min/1.73 m^2^ using the Modification of Diet in Renal Disease study equation. A computed tomography scan of the abdomen and pelvis with intravenous contrast was done. It showed a right renal extracapsular hematoma measuring approximately 4.5 × 9.0 × 15.3 cm^3^ along with an area of hypodensity concerning for extravasation (Figure 1). The size of the right kidney measured 10.4 cm in length, while the left measured 10.9 cm in length.

**Figure 2.**
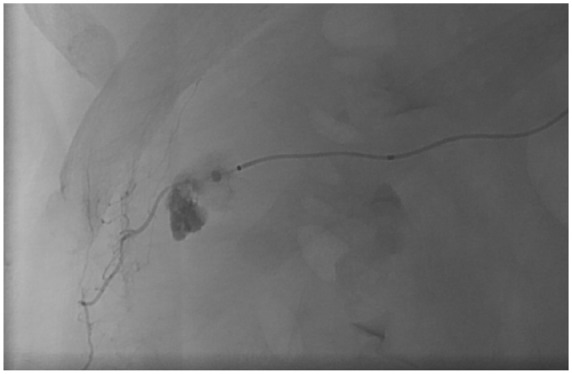
Angiogram of a capsular branch of the right renal artery showing a 2.2 mm intraparenchymal hematoma with active extravasation.

**Figure 3.**
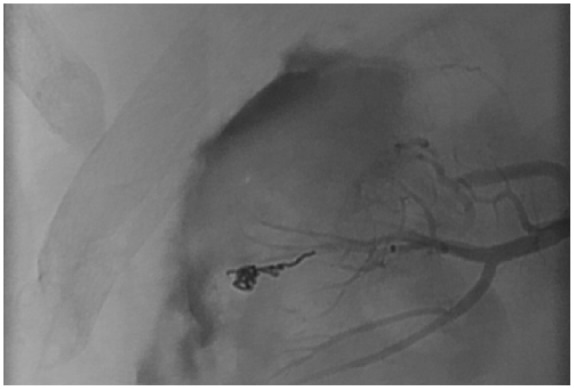
Embolization of the right capsular branch of the renal artery with gelfoam and coils.

**Figure 4.**
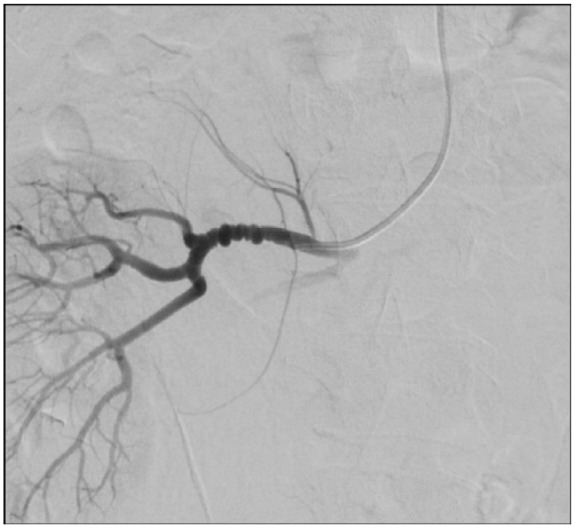
String of beads appearance of the right renal artery characteristic of multifocal fibromuscular dysplasia.

The patient was admitted for observation; however, hemoglobin levels over the next day revealed a drop from 12.9 g/dL (on presentation) to 8.6 g/dL. A catheter-based renal angiogram was performed that revealed a 2.2 mm intraparenchymal hematoma with active extravasation from a capsular branch of the right renal artery. The artery was successfully embolized using gelfoam and coils (Figures 2 and 3). Bilateral irregular beadings in the mid to distal renal arteries were also noted that was compatible with FMD (Figure 4). The differential diagnosis underlying the hematoma includes a pseudoaneurysmal formation secondary to spontaneous rupture of the capsular branch, or a true small aneurysm that has ruptured spontaneously. The patient was discharged home on lisinopril 40 mg and nifedipine XL 60 mg. Her creatinine on discharge was 0.77 mg/dL. She was advised to follow-up with outpatient nephrology for further management. Antiplatelet agents were not prescribed on discharge as the patient did not have additional risk factors, in addition to lack of supportive evidence for their use.^8^

## Discussion

Spontaneous rupture of the renal artery is a rare but documented event, and is usually preceded by pseudoaneurysmal formation.^9,10^ The underlying etiology behind it can vary and has been reported to occur in various clinical scenarios including angiomyolipomas, renal cell carcinoma, acquired cystic kidney disease secondary to hemodialysis, a complication of surgery, or percutaneous procedures or preceded by an RAA.^10-16^

RAAs on the other hand can be either congenital or acquired. They are considered rare in the general population and have been associated with multiple etiologies including tuberous sclerosis, autosomal dominant polycystic kidney disease, malignancy, polyarteritis nodosa, angiomyolipomas, atherosclerosis, and long-standing uncontrolled hypertension and FMD.^17^ There has been controversy over the criteria for RAA repair within the past decades. Usually, for asymptomatic patients without refractory hypertension, the indications agreed upon for repair is a size of more than 2 cm.^18,19^

Our patient had spontaneous rupture of a capsular branch of the renal artery. She was also found to have “string of beads” appearance, which represents alternating areas of stenosis secondary to fibrous webs and poststenotic dilation.^20^ This is characteristic of multifocal FMD, the most common histological type of FMD that is also called medial fibroplasia.^20^ It is unclear whether this spontaneous rupture happened secondary to FMD solely or whether it was preceded by an RAA. If we propose that this event was preceded by an RAA formation secondary to FMD and presumably long-standing hypertension, then the aneurysm is most likely smaller than 2 cm. This might suggest that RAAs associated with FMD could be at an increased risk of rupture even if they are very small. Therefore, they might have a different natural history than RAAs associated with other causes. Nevertheless, and to our knowledge, if we consider both scenarios, this is the first case report of spontaneous renal artery hemorrhage associated with FMD without an obvious large aneurysm.
